# Fermented Garlic Extract Increases Oxygen Consumption and UCP-1 mRNA Expression in Human Adipose-Derived Stem Cells

**DOI:** 10.22074/cellj.2019.6382

**Published:** 2019-06-15

**Authors:** Eulsoon Park, Seung-Hwa Baek, Keuk-Su Bang, Na-Hyung Kim, Koichi Takimoto

**Affiliations:** 1 Department of Bioengineering, Nagaoka University of Technology, Nagaoka, Niigata, Japan; 2 Department of Biofood Science and Biotechnology, Chungbuk Provincial University, Okcheon-gun, Chungbuk, Republic of Korea; 3 Department of Oriental Medicine Resources, College of Environmental and Bioresource Science, Chonbuk National University, Ik-san City, Chonbuk, Republic of Korea; 4 Research Institute of HtO Life, HtO Life Co., LTD, Wanju-gun, Republic of Korea

**Keywords:** Adipose-Derived Stem Cells, Garlic, Mitochondrial Uncoupling Protein, Oxygen Consumption, Thermogenesism

## Abstract

Fermented garlic, often called black garlic, is a traditional food ingredient used in Asian cuisine and possesses various health benefits including anti-obesity activity. The anti-obesity effects of fermented garlic might, in part, might be mediated through direct actions of its components on adipocytes. To test this hypothesis, we examined whether fermented garlic extract might stimulate the metabolic activity of human adipose-derived stem cells (ADSCs) in culture. Cell viability measured by 3-(4,5-dimethylthiazol-2-yl)-2,5-diphenyltetrazolium (MTT) assay exhibited a complex dose- response relationship. The lowest concentration (0.4 mg/ml) reduced cell viability (P<0.05 compared to no extract, Bonferroni’s multiple comparison), whereas higher concentrations (0.8 and 1.0 mg/ml) resulted in higher cell viability (P<0.05 as compared to 0.4 mg/ml). However, the extract at concentrations >2 mg/ml markedly decreased cell viability. Higher cell viability observed following treatment with 0.8~1.0 mg/ml might be associated with raised oxygen consumption. Fluorescent dye-based measurement revealed that the garlic extract at 1.0 mg/ml significantly increased oxygen consumption. We also detected a significant increase in mRNA expression levels of uncoupling protein-1 (UCP- 1). These findings suggest that fermented garlic stimulates the basal metabolic activity of human ADSCs.

## Introduction

Black garlic is produced by fermentation under high temperature and humidity. As it possesses unique texture and flavor and lacks offensive smells, it has become a popular ingredient not only in Asian cuisines, but for other cooking styles of the world. In addition, black garlic has been shown to produce various health benefits, some of which may not be seen with fresh product (1, 2). For example, dietary consumption of black garlic decreased body weight and fat accumulation in a rodent model of obesity (3, 4). Similarly, methanol extract of black garlic reduced fat masses and altered expression of various genes involved in lipid metabolism (5). Notably, the abovementioned changes were observed in the absence of any decline in total food consumption. Therefore, components of black garlic may stimulate basal metabolic activity *in vivo*.

Nonshivering thermogenesis accounts at least in part for alterations in the basal metabolic rate under physiological conditions. Brown adipose tissues are primarily responsible for nonshivering thermogenesis in rodents and human infants. Studies conducted during the last ten years have established that adult humans also possess active brown adipose tissues (6- 8). Furthermore, cells present in white adipose tissues of adult humans become thermogenic under certain conditions (9-11). Considering the alarming increase in the prevalence of obesity and its associated diseases, browning of white fats has gained much attention as a possible therapeutic approach against these detrimental conditions.

Known in vivo health benefits of black garlic consumption are likely to influence various organs and complex physiological mechanisms. It is also possible that black garlic possibly exerts direct beneficial actions on adipocytes. To test this hypothesis, we used human adipose-derived stem cells (ADSCs) and fermented garlic, a type of black garlic that is prepared under sterile and controlled conditions (12). In this study, we found that fermented garlic extract stimulates the basal metabolic activity and browning of human ADSCs.

Ordinary black garlic used for culinary purposes is prepared by fermenting unpeeled garlic using supplemented or naturally-occurring microorganisms. The preparing procedure significantly differs from one region to another and various microorganisms present in the producing environment may potentially affect the final product. Thus, we used fermented garlic prepared under conditions with minimized contamination caused by environmental microorganisms from HtO Life Co., Ltd. (Wanju-gun, Republic of Korea). This product was prepared by fermentation of peeled and crushed garlic cloves. Briefly, peeled garlic cloves were sterilized using ozonized water and crushed into a paste-like preparation. The garlic paste was then fermented using the strain, Bacillus subtilis subsp. subtilis KACC91554P, under aerobic conditions. Water-soluble extract was collected by ultrafiltration, and hot air-dried using a spray dryer (Eyela SD- 1000, Eyela, Japan). The prepared fermented garlic was tested for various components at Namhae Garlic Research Institute (Namhae, Republic of Korea) to ensure the consistency of the preparations (12).

The dried fermented garlic extract was re-dissolved in water at a final concentration of 100 mg/ml by rigorous vortexing and sonication. Undissolved materials were eliminated by centrifugation, followed by filtration through a cellulose acetate membrane (pore size 0.2-mm). The concentrations of fermented garlic mentioned in this paper indicate those measured based on the initial dried powder without considering the undissolved and eliminated materials.

Human adipose-derived stem cells (ADSCs) were purchased from Cellular Engineering Technologies (Coralville, IA, USA). Cells were cultured in Dulbecco’s Modified Eagle Medium supplemented with 10% fetal bovine serum (Gibco, New Zealand), 50 U/ml penicillin and 50 μg/ml streptomycin (Nacalai Tesque, Japan) under 5% CO_2_ atmosphere at 37˚C.

Human ADSCs were seeded in a 96-well dish at ~5000 cells/well. Two days after seeding, cells were treated with fermented garlic extract or water for additional 2 days. Cell viability was then determined using 3-(4,5-dimethylthiazol-2-yl)-2,5-diphenyltetrazolium bromide (MTT) according to the manufacture’s protocol (Thermo Fisher Scientific, Walthum, MA, USA).

Total cellular oxygen consumption was determined by measuring extracellular molecular oxygen using a phosphorescent oxygen-sensitive dye (Abcam, UK). Human ADSCs were treated with 1.0 mg/ml fermented garlic extract or water (1/100 volume) for 2 days. Cells were then subjected to oxygen consumption assays according to the manufacturer’s protocol. Fluorescence was monitored every 1.5 minutes over 90 minutes at 37˚C by a plate reader (Tecan, Switzerland). A linear portion of blank-collected fluorescent intensity in each sample was used to estimate the oxygen consumption rate. Antimycin A, an inhibitor of the electron transport chain, almost completely eliminated the time-dependent increase in fluorescent intensity.

Total RNAs were isolated from cultured human ADSCs using a phenol-based reagent (Sepasol, Nacalai Tesque, Japan) and their concentrations were estimated using absorbance at 260 nm=40 ng/ml. First-strand cDNAs were synthesized using 0.1 mg total RNA with a mixture of oligo(dT) and random primers (ReverTra Ace Master Mix, Japan). PCR was carried out using synthesized cDNA sample (0.1 to 1.0 ml) using primers (Table 1) under the following conditions: denaturation at 95˚C for 5 seconds, annealing at 58˚C for 5 seconds, and extension at 72˚C for 1 minute for 24~30 cycles. Sample volume and cycle numbers were varied for different primer sets and samples. PCR products were separated by a 1.2% agarose gel and stained with ethidium bromide. We semi-quantitatively estimated relative mRNA levels by measuring ethidium bromidestained band intensities using a CCD camera-based imaging system (UVP, Upland, CA).

**Table 1:** Primers used in this study


Name	Primer sequence (5ˊ-3ˊ)	Position	Length	GenBank accession

UCP-1	F: AGGAGTGGCAGTATTCATTGG	448-686	239	NM_021833
	R: TCACAAAGGCCTCCTTCATTAG			
PPARGC1A	F: CAGCTCCAAGACCAGGAAAT	1474-1696	223	NM_013261
	R: CCCAAGGGTAGCTCAGTTTATC			
PPARG	F: GCTGGCCTCCTTGATGAATAA	1234-1438	205	NM_138712
	R: GCGGTCTCCACTGAGAATAATG			
GAPDH	F: GTCAACGGATTTGGTCGTATTG	124-262	139	NM_002046
	R: CATGGGTGGAATCATATTGGAA			


**Fig.1:**
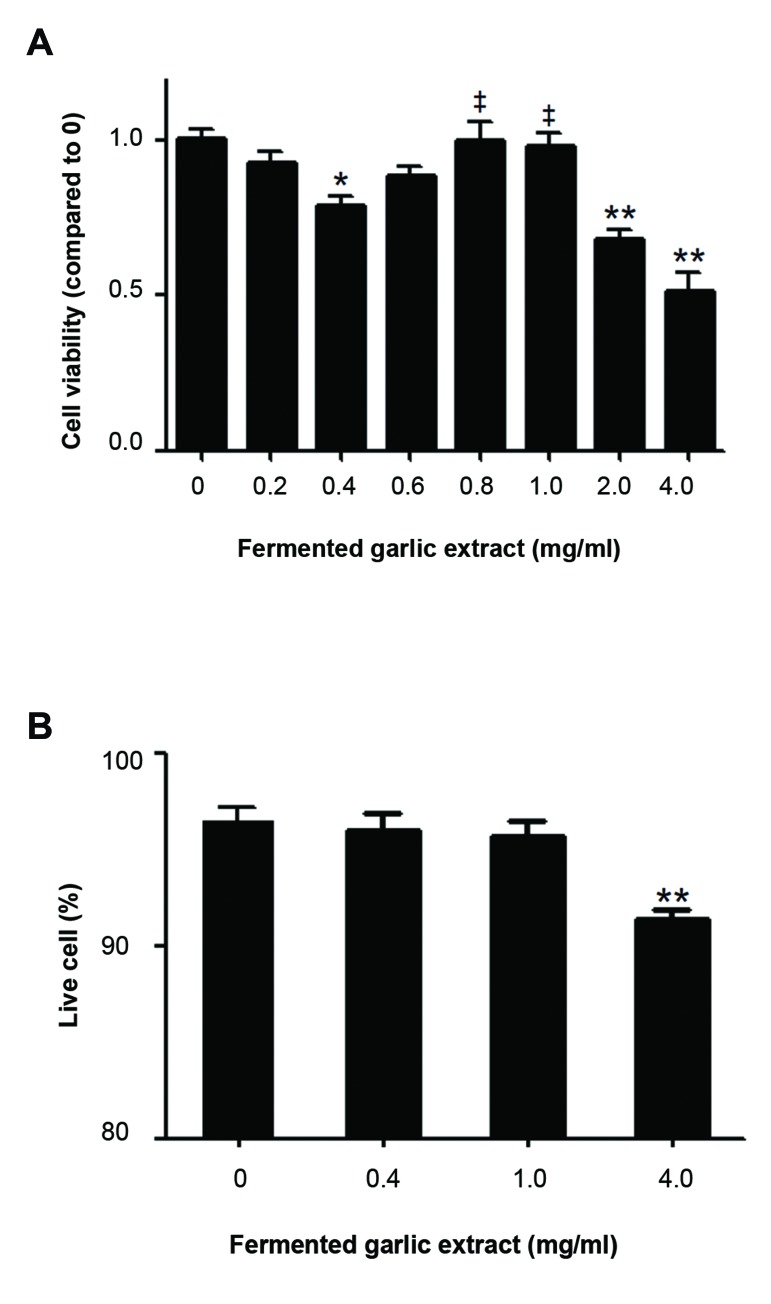
Fermented garlic extract produces a complex dose-response change in human adipose-derived stem cells (ADSC) viability. Human ADSCs were cultured in the presence of black garlic extract at indicated concentrations for 2 days. A. Cell viability was determined using MTT assays (n>18 from at least three independent cell preparations). *; P<0.05, **; P<0.01 show significant differences as compared to no extract (0 mg/ml), whereas‡; P<0.05 shows significant differences as compared to 0.4 mg/ml (oneway ANOVA, followed by Bonferroni’s multiple comparison was used for data analysis) and B. Live cell percentages were determined using trypan blue exclusion. **; P<0.01 show significant differences as compared to no extract (0 mg/ml) (n>6 from two independent cell preparations).

Fermented garlic contains various chemicals that may influence cellular metabolism in distinct ways. We first used MTT assay which is based on cellular metabolic activity in terms of reducing MTT. The MTT assay results should represent total cellular metabolic activity or cell viability in the sample. These assays are widely used to measure cell proliferation and/or chemical’s toxicity. We treated human ADSCs with fermented garlic extract at various concentrations for 2 days and determined cell viability (Fig .1A). The cell viability showed a complex dose-response relationship. At concentrations as low as 0.4 mg/ml, the extract reduced cell viability. However, the extract at slightly higher concentrations, 0.8 and 1.0 mg/ml, caused a regain in the viability. Further increasing the concentration to >2 mg/ml resulted in marked reductions in the viability. Since black garlic extract might influence cell growth or induce cell toxicity, we also determined live cell numbers and percentages using trypan blue exclusion following treatment with several concentrations of the extract (Fig .1B). No significant changes in the live cell number or percentages were detected at the extract concentrations up to 1.0 mg/ml (live cell percentages are shown in Figure 1B, data not shown for live cell numbers). At the highest concentration (4.0 mg/ml) in the current study, the live cell number and percentage slightly decreased (less than 10% of total cells). Thus, the observed rise in cell viability at concentrations around 1.0 mg/ml may be due to the presence of a component in fermented garlic extract that enhances metabolic activity of human ADSCs.

**Fig.2:**
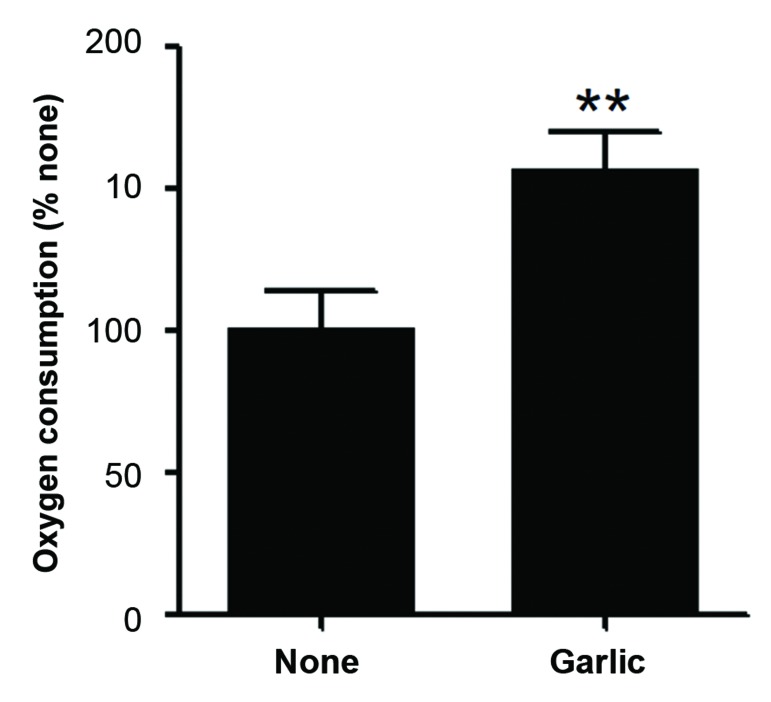
Fermented garlic extract increases cellular oxygen consumption of human adipose-derived stem cells (ADSCs). Human ADSCs were treated with 1.0 mg/ml fermented garlic extract (garlic) or vehicle (1/100 volume of water, none) for 2 days. Oxygen consumption was determined by measuring changes in molecular oxygen in the culture medium with a phosphorescent dye (n=8 from three independent cell preparations). **; P<0.01 shows significant differences as compared to none (t test was used for data analysis).

We wished to further corroborate the possibility that fermented garlic extract stimulates the metabolic activity of ADSCs. Since MTT assays may be associated with off-target effects, we measured cellular oxygen consumption using a molecular oxygensensitive dye (Fig .2). Cellular oxygen consumption was significantly higher in cells treated with 1.0 mg/ ml of the extract for 2 days than vehicle (water)-treated cells. Given that the treatment with fermented garlic extract at this concentration did not influence live cell number or percentage, these results further support the possibility that the extract enhances the metabolic activity of ADSCs.

**Fig.3:**
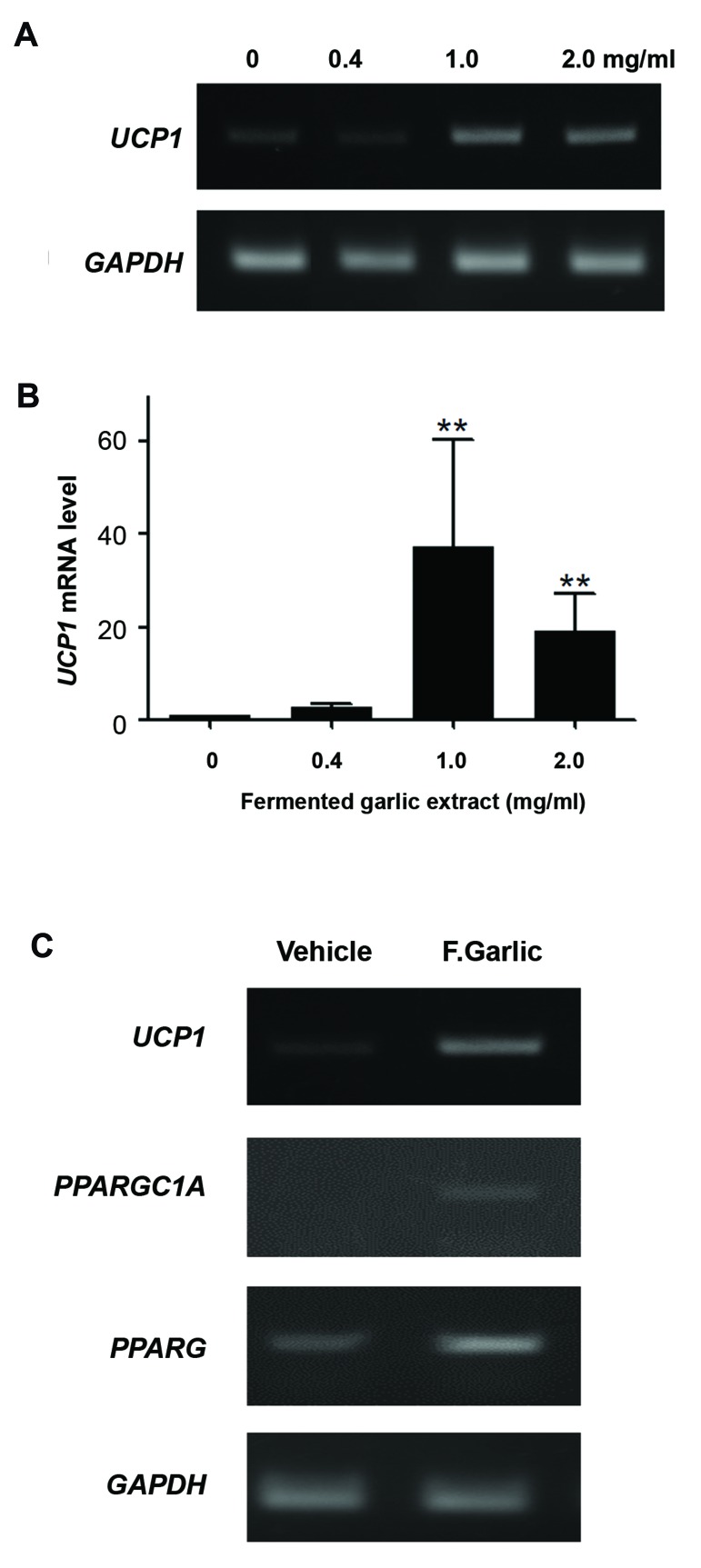
Fermented garlic extract increases expression of UCP1 and brown adipocyte marker mRNAs. Human adipose-derived stem cells (ADSCs) were treated with fermented garlic extract at indicated concentrations for 2 days. Reverse transcription-polymerase chain reaction (RT-PCR) was performed with primers for UCP1, PPARGC1A, PPARG or GAPDH. A. Representative PCR results for UCP-1 mRNA expression, B. UCP-1 mRNA levels were semi-quantitatively estimated using GAPDH mRNA level as an internal control (n=3 from three independent cell preparations). **; P<0.01 shows significant differences as compared to 0 mg/ml (one sample test), and C. Human ADSCs were treated with 0.1 mg/ml fermented garlic extract (F. Garlic) or water (vehicle) for 2 days. Representative PCR data show increased expression of PPARGC1A and PPARG mRNAs, as well as UCP1 transcript.

Then we examined whether the observed increase in oxygen consumption is associated with the browning of human ADSCs. We measured mitochondrial uncoupling protein-1 (*UCP-1*) mRNA levels; *UCP-1* is a key protein in proton leak from the mitochondrial inner membrane and physiological heat generation (Fig .3A, B). Low levels of *UCP-1* mRNA were detected in most samples that were not treated with fermented garlic extract. Importantly, *UCP-1* mRNA level markedly increased following treatment with 1.0 or 2.0 mg/ml of the extract for 2 days. Thus, the *UCP-1*-based proton leak may in part mediate the fermented garlic extract-induced increase in metabolic activity of human ADSCs. We also tested whether fermented garlic might increase mRNA levels for peroxisome proliferator-activated receptor-γ (*PPARG*) and its coactivator-1α (*PPARGC1A*), master regulators of the *UCP-1* gene transcription and mitochondrial biogenesis (Fig .3C). Treatment with fermented 1.0 mg/ml garlic extract for 2 days raised *PPARG* and *PPARGC1A* mRNA expression. These findings suggest that fermented garlic induces the browning of human ADSCs.

Fermented or black garlic has been shown to produce various health benefits. In particular, intake of black garlic reduces body weight and fat masses, and normalizes physiological and biochemical parameters in animal models of obesity (3-5). However, it remains unknown whether component(s) in these fermented garlic products act directly on adipocytes to produce any beneficial changes. In this paper, we showed that fermented garlic extract increases oxygen consumption and *UCP-1* mRNA level in cultured human ADSCs. In addition, the extract increased mRNA levels of *PPARγ* and *PGC-1α* that play pivotal roles in *UCP-1* expression and mitochondrial biogenesis in adipocytes (13). Thus, component(s) present in fermented garlic may directly activate thermogenesis of these adult body-residential cells.

It has become evident that cells located in white adipose tissues of adult rodents (14-17) and humans (9-11) become thermogenic under certain conditions. Moreover, prolonged cold exposure or treatment with b3 agonists converts white fats to brown fat-like heatgenerating tissues in intact animals (18, 19). These brown adipocyte-like cells possess gene expression profiles that are distinct from those of standard brown adipocytes and are called “brite” or “beige” adipocytes (20, 21). The overweight population has tripled in the last 40 years in the world; also, obesity has appeared as a major risk factor for various diseases. Therefore, inducing browning of white adipose tissue-residential cells is considered to hold a promising therapeutic potential against this major health problem. Our finding which showed that fermented garlic, a simple food ingredient, can stimulate this process, may provide basis for economical interventions for the prevention of obesity. Additionally, fermented or black garlic is known to enhance food flavor, and may be easily introduced to diverse cooking styles.

Fermented or black garlic contains various chemical components. Unlike fresh garlic, the sulfur-containing alliin and its converted substances with offensive flavors are much less abundant in fermented products (1, 2, 22). In contrast, the main sulfur-containing product appears to be S-allylcysteine in aged or fermented garlic products. In addition, polyphenols, flavonoids, and several compounds generated by the Amadori and Heyns rearrangements are found at much higher levels in these garlic products (1, 2, 23, 24). Some of these compounds were shown to possess biological activities. For example, S-allylcysteine possesses antioxidant and anti-inflammatory activities (25). Likewise, polyphenols in aged or fermented garlic are proposed to contribute to antioxidant properties of these products (26). In this study, we used total water extract of fermented garlic. This preparation yielded a complex dose-response change in MTT assays, likely due to the presence of various components and their potential interactions. Thus, it would be certainly important to identify component(s) present in this preparation that mediate(s) the observed stimulation of oxygen consumption and UCP-1 mRNA expression. Taken together, further identification of the chemical component(s) responsible for the observed effects, as well as molecular mechanistic studies, may yield novel and useful information on the use of fermented garlic for prevention and treatment of obesity and its associated diseases.
